# Supplementary material for: Interpersonal alignment of neural evidence accumulation to social exchange of confidence
Source: eLife. 2023 Dec 21;12:e83722. doi: 10.7554/eLife.83722 (PMC10746141; doi:10.7554/eLife.83722)
Supplement: Supplementary file 1. [file elife-83722-supp1.docx]

**Supplementary Information for**

**Interpersonal alignment of neural evidence accumulation to social exchange of confidence**

Jamal Esmaily, Sajjad Zabbah, Reza Ebrahimpour, Bahador Bahrami

Corresponding authors:

Jamal Esmaily (email: [jamal.esmaily@campus.lmu.de](mailto:jamal.esmaily@campus.lmu.de))

Reza Ebrahimpour (e-mail: [rebrahimpour@sru.ac.ir](mailto:rebrahimpour@sru.ac.ir))

Bahador Bahrami (e-mail: [bbahrami@gmail.com](mailto:bbahrami@gmail.com))

1. **Supplementary Tables**

**Table a.** details of statistical results in behavioral data (figure 1):

|  | **Response** | **Regressors** | **Estimate** | **SE** | **CI** | **tStat** | **pvalue** | **Total Number** |
| --- | --- | --- | --- | --- | --- | --- | --- | --- |
| **Study 1** | **Accuracy**  **(HC vs LC)** | **Coherency** | 0.007 | 0.0006 | [.006 .008] | 11.57 | <.001 | 9600 |
|  |  | **Condition** | -0.002 | 0.021 | [-.045 .04] | -0.1 | .92 | 9600 |
|  | **Confidence**  **(HC vs LC)** | **Coherency** | 0.0475 | 0.0008 | [.046 .049] | 56.5 | <.001 | 9600 |
|  |  | **Condition** | 1.361 | 0.03 | [1.31 1.42] | 46.4 | <.001 | 9600 |
|  | **RT**  **(HC vs LC)** | **Coherency** | -0.005 | 0.0001 | [-.005 -.004] | -44.4 | <.001 | 9600 |
|  |  | **Condition** | 0.029 | 0.004 | [-.035 -.021] | 7.85 | <.001 | 9600 |
| **Study 2** | **Accuracy**  **(HC vs LC)** | **Coherency** | 0.0209 | 0.0016 | [.017 .024] | 13.23 | <0.001 | 6000 |
|  |  | **Condition** | -0.0092 | 0.0296 | [-.067 .049] | -0.31 | .76 | 6000 |
|  | **Confidence**  **(HC vs LC)** | **Coherency** | 0.1011 | 0.1011 | [.097 .106] | 47.47 | <0.001 | 6000 |
|  |  | **Condition** | 0.496 | 0.037 | [.42 .56] | 13.32 | <0.001 | 6000 |
|  | **RT**  **(HC vs LC)** | **Coherency** | -0.009 | 0.0003 | [-.01 -.008] | -26.22 | <0.001 | 6000 |
|  |  | **Condition** | 0.0363 | 0.006 | [.024 .048] | 6.12 | <0.001 | 6000 |

**Table b.** Details of statistical results in pupil data (figure 2)

|  | **Response** | **Regressors** | **Estimate** | **SE** | **CI** | **tStat** | **pvalue** | **Total Number** |
| --- | --- | --- | --- | --- | --- | --- | --- | --- |
| **Study 1** | **Pupil** | **Condition** | -0.038 | 0.011 | [-.06 -.01] | -3.30 | <.001 | 8390 |
| **Study 2** | **Pupil** | **Condition** | -0.066 | 0.015 | [-.09 -.04] | -4.37 | <.001 | 5842 |

**Table c:** Details of statistical results in EEG data (figure 4)

|  | **Response** | **Regressors** | **Estimate** | **SE** | **CI** | **tStat** | **pvalue** | **Total Number** |
| --- | --- | --- | --- | --- | --- | --- | --- | --- |
| **Study 1** | **EEG Slope** | **Coherency** | 0.62 | 0.065 | [.49 .74] | 9.64 | <.001 | 6492 |
|  |  | **Condition** | 0.2 | 0.14 | [.-.07 .49] | 1.42 | .15 | 6492 |
| **Study 2** | **EEG Slope** | **Coherency** | 0.8 | 0.29 | [.24 1.37] | 2.8 | <.01 | 5367 |
|  |  | **Condition** | 1.52 | 0.63 | [.27 2.77] | 2.39 | .017 | 5367 |

**Table d:** Details of statistical results in EEG data (figure S8 top row)

|  | **Response** | **Regressors** | **Estimate** | **SE** | **CI** | **tStat** | **pvalue** | **Total Number** |
| --- | --- | --- | --- | --- | --- | --- | --- | --- |
| **Study 1** | **EEG Slope** | **Coherency** | 0.02 | 0.005 | [0.01 0.03] | 4.48 | <.001 | 1523 |
| **Study 2** | **EEG Slope** | **Coherency** | 0.06 | 0.02 | [0.01 0.11] | 2.54 | <.01 | 2822 |

**Table e.** details of statistical results in for the impact of previous trial:

|  | **Response** | **Regressors** | **Estimate** | **SE** | **CI** | **tStat** | **pvalue** | **Total Number** |
| --- | --- | --- | --- | --- | --- | --- | --- | --- |
| **Study 1** | **Accuracy**  **(HC vs LC)** | **Coherency** | 0.007 | 0.0006 | [.006 .008] | 11.58 | <.001 | 9600 |
|  |  | **Conf (t-1)** | -0.0017 | 0.005 | [-.01 .01] | -0.28 | .77 | 9600 |
|  | **Confidence**  **(HC vs LC)** | **Coherency** | 0.047 | 0.001 | [.045, .049] | 54.7 | <.001 | 9600 |
|  |  | **Conf (t-1)** | 0.32 | 0.008 | [.3 .33] | 38.31 | <.001 | 9600 |
|  | **RT**  **(HC vs LC)** | **Coherency** | -0.005 | 0.0001 | [-.0048 .0044] | -44.36 | <.001 | 9600 |
|  |  | **Conf (t-1)** | -0.0055 | 0.001 | [-.007 -.003] | -5.44 | <.001 | 9600 |
| **Study 2** | **Accuracy**  **(HC vs LC)** | **Coherency** | 0.02 | 0.002 | [0.02 .024] | 13.23 | <0.001 | 6000 |
|  |  | **Conf (t-1)** | .003 | .008 | [-.012 .018] | 0.37 | .7 | 6000 |
|  | **Confidence**  **(HC vs LC)** | **Coherency** | 0.1 | .002 | [.097 .0106] | 47.2 | <0.001 | 6000 |
|  |  | **Conf (t-1)** | 0.09 | 0.01 | [.07 .11] | 8.6 | <0.001 | 6000 |
|  | **RT**  **(HC vs LC)** | **Coherency** | -0.009 | 0.0003 | [-.001 -.008] | -26.2 | <0.001 | 6000 |
|  |  | **Condition** | 0.005 | 0.001 | [.001 .008] | 2.98 | <0.01 | 6000 |

**Table f:** the rate of trial rejection of eye tracking (only data of social) and EEG data (visual inspection) per participant

|  | Participants | Eye Tracking Rejection % (Social) | EEG Trial Rejection % (Visual) |
| --- | --- | --- | --- |
| study 1 (Discovery) | 1 | 12.25 | 4.6 |
|  | 2 | 12.87 | 31.1 |
|  | 3 | 0.5 | 22.1 |
|  | 4 | 4 | 14.8 |
|  | 5 | 1.37 | 34.4 |
|  | 6 | 0 | 4.6 |
|  | 7 | 7.75 | 8.8 |
|  | 8 | 0.37 | 24.4 |
|  | 9 | 6.37 | 7.6 |
|  | 10 | 0 | 46 |
|  | 11 | 0.12 | NA |
|  | 12 | NA | NA |
| study 2 (Replication) | 1 | 0 | 4 |
|  | 2 | 1.25 | 1 |
|  | 3 | 5.75 | 8.5 |
|  | 4 | 0.5 | 3 |
|  | 5 | 1 | 16 |
|  | 6 | 1.5 | 2.5 |
|  | 7 | 0 | 0.5 |
|  | 8 | 1.5 | 9 |
|  | 9 | 0 | 2 |
|  | 10 | 1 | 4 |
|  | 11 | 1 | 7.5 |
|  | 12 | 0.5 | 0 |
|  | 13 | 0.75 | 10.5 |
|  | 14 | 2.5 | 12 |
|  | 15 | 14.75 | 4.5 |

**Table g: GLMM model including interaction terms (p-values are reported)**

|  | **Response** | **Coherence** | **Condition (LC vs HC)** | **Condition * Coherence** |
| --- | --- | --- | --- | --- |
| **Study 1** | **Accuracy** | p<0.001 | p=0.92 | p=0.96 |
|  | **Confidence** | p<0.001 | p<0.001 | p<0.001 |
|  | **RT** | p<0.001 | p<0.001 | p<0.05 |
|  | **Pupil** | p=0.43 | p=0.20 | P=0.31 |
|  | **EEG Slope** | p<0.01 | p=0.15 | p=0.91 |
| **Study 2** | **Accuracy** | p<0.001 | p=0.75 | p=0.87 |
|  | **Confidence** | p<0.001 | p<0.001 | p<0.001 |
|  | **RT** | p<0.001 | p<0.001 | p=0.34 |
|  | **Pupil** | p=0.35 | p=0.06 | p=0.17 |
|  | **EEG Slope** | p=0.62 | p<0.05 | p=0.68 |

**Table h: attractor model’s parameters**

| **Reference, remarks** | **Parameter value** | **Parameter** |
| --- | --- | --- |
| Calibrated based on pool of Isolated data, also fitted on individual subjects’ data | 0.3157 nA | JN,ii |
| Calibrated based on pool of Isolated data, also fitted on individual subjects’ data | 0.0646 nA | JN,ij |
| Calibrated based on pool of Isolated data, also fitted on individual subjects’ data | 45.8 Hz | µ0 |
| Calibrated based on pool of Isolated data, also fitted on individual subjects’ data | 0.27 s | NDT |
| Calibrated based on pool of Isolated data, also fitted on individual subjects’ data | 0.32 nA | Bound |
| Calibrated based on pool of Isolated data, also fitted on individual subjects’ data | -0.99 | a (equation 9) |
| Calibrated based on pool of Isolated data, also fitted on individual subjects’ data | 1.32 | b0 (equation 9) |
| Calibrated based on pool of Isolated data, also fitted on individual subjects’ data | -0.165 | b1 (equation 9) |
| Calibrated based on pool of Isolated data, also fitted on individual subjects’ data | 5.9 | k (equation 9) |
| From (Wang, 2002; Wong, 2006) | 0.3255 nA | I0 |
| From (Wang, 2002; Wong, 2006) | 0.00022 nA Hz-1 | JA.ext |
| From (Wang, 2002; Wong, 2006) | 0.1 s | τs |
| From (Wang, 2002; Wong, 2006) | 0.0005 s | dt |
| From (Wang, 2002; Wong, 2006) | 270 (V nC)-1 | a (equation 7) |
| From (Wang, 2002; Wong, 2006) | 108 Hz | b (equation 7) |
| From (Wang, 2002; Wong, 2006) | 0.154 s | d (equation 7) |
| From (Wang, 2002; Wong, 2006) | 0.641 | γ |
| From (Wang, 2002; Wong, 2006) | 0.025 | Noise_std |
| From (Wang, 2002; Wong, 2006) | 0.02 | I_noise |
